# Aligner therapy in adolescents: first-year results on the impact of therapy on oral health-related quality of life and oral hygiene

**DOI:** 10.1007/s00784-022-04741-1

**Published:** 2022-10-29

**Authors:** Mareike Kristin Sauer, Thomas Drechsler, Priscilla Ferrari Peron, Irene Schmidtmann, Daniela Ohlendorf, Heinrich Wehrbein, Christina Erbe

**Affiliations:** 1 Department of Orthodontics, University Medical Center of the Johannes Gutenberg-University, Augustusplatz 2, 55131 Mainz, Germany; 2 Orthodontic Practice, Wilhelmstraße 40, 65183 Wiesbaden, Germany; 3 Institute for Medical Biostatistics, Epidemiology and Informatics (IMBEI), University Medical Centre of the Johannes Gutenberg-University, Mainz, Germany; 4 Institute for Occupational Medicine, Social Medicine and Environmental Medicine, Goethe-University, Frankfurt am Main, Germany

**Keywords:** Quality of life, Adolescents, Aligner therapy, Oral hygiene

## Abstract

**Objectives:**

The aim of this multicenter clinical study was to examine the oral health-related quality of life and oral hygiene in adolescents before and during aligner therapy.

**Materials and methods:**

Forty subjects (18 ♀, 22 ♂; mean age: 13.6 years) scheduled for aligner therapy (Invisalign® Teen) were given oral health-related quality of life questionnaires, Oral Health Impact Profile (OHIP-G14) and Psychosocial Impact of Dental Aesthetic Questionnaire (PIDAQ), to complete within their treatment (visit 1: 0 start of therapy; visit 2: 0 + 4 weeks; visit 3: 0 + 10 weeks; visit 4: 0 + 6 months; visit 5: 0 + 1 year). To assess oral hygiene, a questionnaire to take home was used, and plaque level was evaluated with the Quigley-Hein Plaque Index (TMQH) modified by Turesky et al.

**Results:**

The OHIP-G14 mean score before aligner therapy was 3.3 ± 3.2, and 4.9 ± 5.4 after 1 year. The PIDAQ showed a positive psychological change in the well-being, as well as a more effective at-home oral hygiene regime. On average, the TMQH remained at a low level (grade 2 of 0–5). The initial insertion of the aligners caused the most significant changes in all parameters (except TMQH).

**Conclusion:**

Oral health-related quality of life is only slightly affected during the first year of aligner therapy in adolescents. Oral hygiene at home is intensified and there is no increased dental plaque accumulation.

**Clinical relevance:**

Dentofacial esthetics is a subset of the so-called Oral H-Related Quality of Life (OHRQoL) which should be considered more during orthodontic therapy.

## Introduction

It is becoming increasingly common for patients to select orthodontic therapies based solely on esthetic considerations [1–4]. The improvements seen from orthodontic therapy result in an increased quality of life, thus motivating patients to undergo treatment [3, 5].

The desire for an esthetically pleasing orofacial appearance and an increased quality of life associated with it results in a growing interest in low-profile orthodontic appliances for the regulation of tooth and jaw malocclusions [6–10]. Removable transparent trays made of flexible plastic—so-called aligners—are available to the practitioner as an orthodontic appliance [11, 12]. The orthodontic technique of adjusting teeth with a series of removable, flexible trays dates back to Kesling [13] and the year 1945. The Invisalign® Teen system (Align Technology Inc., Santa Clara, CA, USA) was first introduced in 1997 and has been available in Germany since 2001 [11, 12]. Cross-linking this system with innovative technologies, such as CAD and CAM, simplified orthodontic treatment and resulted in an efficient and economic method for the orthodontic practice [14, 15]. Meanwhile, this technique has also become available for a wide range of orthodontic indications [12].

Children and adolescents represent the main group of patients in orthodontics. During this age range, the periodontium can still be significantly remodeled while making use of patients’ physiological growth [16]. In this age group, adolescents are the most predisposed to develop dental caries and gingivitis during orthodontic therapy [17]. Since orthodontic appliances generate retention areas for the accumulation of dental plaque, oral hygiene instructions are of vital importance [18–20]. Clinical studies have confirmed that a therapy with fixed multibracket appliances leads to a higher risk of gingivitis or enamel demineralization [18, 21–23]. Further clinical trials conclude that treatments with removable aligners (Invisalign® Teen) result in lower dental plaque accumulation rates and better periodontal health when compared to treatments with fixed multibracket appliances [24, 25]. Applying aligners (Invisalign® Teen) for orthodontic treatment rather than multibracket appliances produces less limitations concerning oral health-related quality of life [25, 26].

The objective of this multicenter clinical study was to examine the effect of aligner therapy in a cohort of adolescents (Invisalign® Teen, Align Technology Inc., Santa Clara, CA, USA) on 1.) oral health-related quality of life and 2.) oral hygiene during the first year of treatment.

The presented results are published as an exploratory analysis of 1-year results of a larger ongoing clinical study “Studie zur Untersuchung des Mundhygienestatus und der Lebensqualität des Patienten vor, während und nach der Aligner-Therapie (Invisalign®).” The primary endpoints are OHIP and PIDAQ at 1 year after debonding and will be reported after completion of the study.

## Materials and method

The clinical surveys were performed in the Department of Orthodontics of the University Medical Center at the Johannes Gutenberg-University Mainz and the orthodontic practice of Dr. med. dent. Thomas Drechsler in Wiesbaden. The focus of the larger study, for which sample size calculation was performed using SAS PROC POWER, was to establish a medium effect in three primary endpoints, i.e., an effect size of 0.5 at the 1.67% significance level each (Bonferroni-correction) with 80% power using three paired *t*-tests. This required 45 participants. Assuming a dropout rate of about 18%, we included 55 patients.

So far, 1-year results are available for 40 adolescents scheduled for orthodontic therapy with aligners (Invisalign® Teen) (18 female, 22 male) who were 11 to 17 years old at the time of study start. Subjects and their legal guardians received written information regarding the study procedure and signed informed consent forms prior to study entry. Participants were required to agree not to participate in any other clinical studies for the entire data collection period. They were instructed to have no external professional dental cleanings, to maintain their current brushing habits (manual or electric), and to not clean their teeth the morning prior to the study appointments. The inclusion criteria were 1) general health, 2) at least 16 natural teeth—at least 8 of which were anterior teeth—and 3) vestibular and lingual tooth surfaces sufficiently assessable. Exclusion criteria were 1) lack of indication for orthodontic therapy, 2) expected insufficient compliance, 3) other ongoing orthodontic treatment, 4) previous therapy with multibracket appliance, 5) presence of severe periodontal disease, 6) ongoing periodontal therapy, 7) allergies to dyes or food, 8) more than three carious defects requiring treatment, 9) use of an antibiotic within two weeks prior to study entry, 10) professional dental cleaning within two weeks prior to study entry, 11) having a pacemaker, 12) pregnancy, and 13) syndromic conditions.

Two valid and reliable questionnaires were used to assess oral health-related quality of life: Oral Health Impact Profile (OHIP) and Psychosocial Impact of Dental Aesthetic Questionnaire (PIDAQ) [27, 28]. The German short version of the OHIP with 14 questions (OHIP-G14) was used [29]. This version of the OHIP is based on the original version by Slade [30] and its official short version of 14 questions by John et al. [29] and includes the following domains: “functional limitations,” “pain,” “psychological discomfort/discomfort,” “physical impairment,” “psychological impairment,” “social impairment,” “disadvantage/disability” [31]. Scores are given on a 5-point scale (0 to 4). The sum of individual scores are results between 0 and 56 (“none”/ “maximum impairment of oral health-related quality of life”) [32]. Children and adolescents were given the German-language version of the PIDAQ; the questionnaire for Dental Aesthetic Related Quality of Life (DARQoL) [33]. The 23 statements of the PIDAQ refer to the following subscales: “dental self-confidence,” “social impairment,” “psychological impairment,” and “aesthetic concern.” Scoring is on a 5-point scale (1 to 5), and the resulting sum score lies between 23 and 115 (“no influence”/ “maximum influence of dental esthetics on oral health-related quality of life”) [28]. To ensure a standardized evaluation, the scores of the only positively formulated subscale (“dental self-confidence”) were taken inversely into evaluation. Home oral hygiene behavior was assessed by means of a catalog of 11 items, including the duration and frequency as well as the overall use of dental care and oral health products, designed in the Department of Orthodontics at the University Medical Center Mainz. The items were rated on a 5-point scale (1 to 5). Supragingival dental plaque accumulation was determined using the Quigley-Hein index (TMQH) modified according to Turesky et al. [34]. This distinguishes the following grades: 0 = no plaque, 1 = scattered plaque patches at the marginal edge, 2 = a thin continuous plaque line (up to 1 mm) at the marginal edge, 3 = a plaque line < 1 mm but > 1/3 of the crown, 4 = plaque covering at least 1/3 to < 2/3 of the crown, 5 = plaque covering 2/3 or more of the crown. Teeth with attachments were rated according to the modified version of the Quigley-Hein index according to Kossack and Jost-Brinkmann [35]: 0 = no plaque, 1 = single plaque areas, 2 = appearance of discrete plaque lines, 3 = plaque extension up to one third of the tooth surface, 4 = plaque extension up to two thirds of the tooth surface, 5 = plaque extension more than two thirds of the tooth surface.

The surveys were distributed over the first year of aligner therapy: divided into five visits (visit 1: start of aligner therapy; visit 2: 4 weeks after the start of aligner therapy—attachment of attachments; visit 3: 10 weeks after start of aligner therapy; visit 4: 6 months after start of aligner therapy; visit 5: 1 year after start of aligner therapy). The study sessions preceded each orthodontic appointment. Due to the limited timeframe of 1 year, the visits after the study such as further treatment time, debonding, and follow-ups after debonding are not included in this data. The data collected in visit 1 provided baseline values. A one-time oral hygiene instruction with illustrations was presented during the first visit. At the beginning of each visit, participants were given the questionnaires to complete. This was then always followed by the clinical survey of the TMQH with subsequent professional tooth cleaning.

The following software was used for statistical analysis: SAS 9.4 (SAS Institute, Cary, NC), IBM SPSS 23 (IBM Corp., Armonk, NY, USA), and Microsoft® Excel. Mean imputation was used for incomplete data. Descriptive statistics were drawn from the data. Differences between time points were assessed using Friedman’s test [36]. These tests are exploratory; therefore, *p* values should be interpreted in a descriptive fashion. In this exploratory analysis, no adjustment for multiple testing is performed. *p* values less than or equal to 0.05 are termed significant.

## Results

The collective included a total of 40 adolescents (18 female, 22 male), aged 11 to 17 years (Ø 13.6 years) at the study baseline (Table 1). The number of subjects differed between visits (V1 to V5) due to some missed appointments or incomplete survey, the number of questionnaires to be evaluated varied (V1 = 39; V2 = 40; V3 = 38; V4 = 36; V5 = 33). Results of the TMQH were available for the following numbers of patients: V1 = 40; V2 = 40; V3 = 38; V4 = 36; V5 = 32.

**Table 1:** Age distribution of the test subjects at visit 1

Age in years	Visit 1
11	7
12	6
13	8
14	7
15	3
16	7
17	2

The statistical analysis of the OHIP-G14 data yields a slight increase in the mean over the entire survey period (Table 2). The greatest mean increase is seen between visits 1 and 2—after the first insertion of the aligners. The mean value subsequently decreases again and after 1 year is slightly above the initial value of visit 1.

**Table 2:** Gender independent summary of the OHIP-G14 scores by visit

	Visit 1	Visit 2	Visit 3	Visit 4	Visit 5
Average	3.3	5.7	5.5	5.4	4.9
Median	3	4	3	3.5	4
Standard deviation	3.2	5.5	6.3	5.6	5.4
Minimum	0	0	0	0	0
Maximum	12	22	29	21	24

The gender-specific values present a similar initial mean value (female: 3.1 ± 3.3; male: 3.5 ± 3.1), though that of the female population is slightly higher than that of the males (Table 3).

**Table 3:** Gender specific OHIP-G14 score summary for each visit

	Visit 1	Visit 2	Visit 3	Visit 4	Visit 5
*Male*					
Mean	3.5	5.5	4.9	5.5	4.1
Median	3.0	5.0	3.5	4.0	4.0
Standard Deviation	3.1	4.3	4.2	5.1	3.3
Minimum	0.0	0.0	0.0	0.0	0.0
Maximum	12.0	15.0	16.0	21.0	11.0
*Female*					
Mean	3.1	6.1	6.1	5.3	5.9
Median	2.0	3.0	3.0	3.0	4.0
Standard Deviation	3.3	6.9	8.0	6.3	7.4
Minimum	0.0	0.0	0.0	0.0	0.0
Maximum	12.0	22.0	29.0	19.0	24.0

Evaluation of the PIDAQ showed that the mean value decreased over the observation period (Table 4).

**Table 4:** Gender independent summary of the PIDAQ scores from each visit

	Visit 1	Visit 2	Visit 3	Visit 4	Visit 5
Mean	47.5	44	44.9	43	41.5
Median	45	39.5	40.8	39	36
Standard Deviation	15.1	15.7	16.4	15.9	13.7
Minimum	29	25	23	23	23
Maximum	96	87	95	88	71

The largest mean decrease is seen between visits 1 and 2. The male population presented lower mean values and a greater decrease in the mean value between visit 1 and visit 2 (Table 5).

**Table 5:** Gender specific PIDAQ summary score for each visit

	Visit 1	Visit 2	Visit 3	Visit 4	Visit 5
*Male*					
Mean	46.0	41.4	41.8	40.9	38.7
Median	43.0	36.0	38.5	38.0	36.0
Standard Deviation	15.1	15.1	15.0	14.4	13.0
Minimum	29.0	25.0	23.0	23.0	23.0
Maximum	83.0	81.0	80.0	76.0	62.0
*Female*					
Mean	49.3	47.2	48.3	45.6	45.3
Median	46.5	43.0	42.5	41.0	46.5
Standard Deviation	15.3	16.3	17.6	17.7	14.0
Minimum	34.0	30.0	30.0	25.0	29.0
Maximum	96.0	87.0	95.0	88.0	71.0

After specific evaluation of the subscales from the PIDAQ, it is noticeable that the mean value in the subscale “dental self-confidence” increases, the subscale “social impairment” remains at a relatively constant level, and the mean values of the subscales “mental impairment” and “aesthetic concern” decrease slightly. The descriptive statistics of the data from the Home Oral Hygiene Behavior Questionnaire reveal an increase in the mean, which is most noticeable between visits 1 and 2 (Table 6).

**Table 6:** Gender independent summary score of home oral hygiene in percent for each visit

	Visit 1	Visit 2	Visit 3	Visit 4	Visit 5
Mean	67.1	76.3	76.9	77.3	77.9
Median	68.9	77	76.4	79.1	78.2
Standard Deviation	11.4	9.3	9.8	9.9	9.6
Minimum	33	50	56	58	58
Maximum	87	91	96	96	96

The TMQH documents relatively constant mean index values across visits (Table 7). The Friedman test reveals no differences between the survey time points for both the OHIP-G14 (*p* = 0.106) and the TMQH (*p* = 0.441). However, differences can be established in the case of the PIDAQ (*p* < 0.001).

**Table 7:** Gender independent TMQH for each visit

	Visit 1	Visit 2	Visit 3	Visit 4	Visit 5
Mean	2	1.9	2	2.1	2.1
Median	2	2	2	2.1	2
Standard Deviation	0.5	0.5	0.5	0.5	0.4
Minimum	0.8	0.7	1	1	1.3
Maximum	2.9	3	3.1	2.9	3

## Discussion

This multicenter clinical study aimed to assess the effects of aligner therapy (Invisalign® Teen) on oral health-related quality of life and oral hygiene over the first year of therapy in adolescents. The population was divided into two groups of approximately equal size, 18 female and 22 male subjects. The study included subjects between the ages of 11 and 17 years at the start of therapy, which represents the majority of the orthodontic patient clientele, and yet, this age group has received little attention in previous studies regarding the topic of this study.

Studies on oral health-related quality of life, which compare treatments with aligners to those with fixed appliances, conclude a higher quality of life with aligner therapy [25, 26, 37]. Other authors also conclude that aligner therapy only slightly influences oral health-related quality of life [11, 38].

For this study, the German short version of the OHIP, the so called OHIP-G14, was used. This version is based on the original version by Slade [30] and its official short version of 14 questions [29], therefore, internationally comparable. In the course of developing the OHIP, a population of adults was studied [27].

Critically, it can be questioned why, in the context of the study conducted here on children and adolescents, the specific questionnaires available were not chosen to assess the OHRQoL. The most widely used questionnaire for children and adolescents is the Child Perceptions Questionnaire, which is characterized by the highest validity and reliability [39], or the Child Oral Health Impact Profile—COHIP for short—which is a version of the OHIP for children [40].

Even though available in the versions, for ages 8 to 10 and 11 to 14 [41, 42] and for 8- to 15-year-olds [39], the age groups of the subjects assessed here (11–17 years) could not have been fully captured with the previously mentioned instruments. Therefore, the choice fell on the OHIP, which does not impose any age restrictions.

The OHIP is one of the most widely used instruments for measuring OHRQoL in Europe [43]. Furthermore, it is validated and reliable [27]. In the field of orthodontics, OHIP has recently been widely used, including in adolescents [44–48].

When regarding this scientific work, the evaluation of the OHIP-G14 reveals OHRQoL limitations within the first year of therapy. The most significant restriction of the OHRQoL occurs after the initial insertion of the aligners in the period from visit 1 to visit 2. A decreasing trend is seen in the following visits. After 1 year, the OHRQoL was only slightly reduced in comparison to the study baseline value. Further studies support the conclusion that OHRQoL experiences the greatest limitations at the beginning of orthodontic therapy [49, 50]. Paes da Silva et al. [48] found a total mean OHIP-G14 value of 8.9 ± 7.3 for a group of adolescents (12 to 17 years) undergoing orthodontic therapy with fixed or removable appliances. Kang and Kang [49] used the 14-question OHIP to conclude a total mean score of 18.39 ± 8.01 for adult subjects (18 to 39 years) undergoing fixed therapy. Female subjects were found to be more impaired in their OHRQoL. In the present study, the mean score at each point in time is relatively low considering the possible ranges for the total score of OHIP G14 (0 to 56); this is confirmed through comparison with previously cited studies. Furthermore, gender differences do not emerge clearly in the present study. The Friedman test does not identify any statistical differences between survey time points for the OHIP-G14 data. This leads to the conclusion that the OHRQoL does not experience any statistically provable limitations over the survey period selected here.

For the present study, it was relevant to choose two kinds of measurement instruments with different focus to elicit OHRQoL. This was to include a wide range of aspects of OHRQoL. The OHIP is also used in general dentistry, whereas the PIDAQ was developed specifically for the survey in orthodontics. The German short version of the OHIP with 14 questions (OHIP-G14) and the PIDAQ in a modified version for children and adolescents were used. The two questionnaires for the assessment of OHRQoL complement each other in terms of content. The OHIP measures the social influence of oral diseases or, in the case of orthodontics, dysgnathic dentition, while the PIDAQ measures the psychosocial effects of dental esthetic appearance.

The data obtained with the PIDAQ makes it clear that the OHRQoL of the subjects is positively influenced over the course of the first year of therapy. This is particularly true for the male subjects since they have continuously lower mean values than the female subjects. After insertion of the aligners, an increase in psychosocial well-being can be observed across genders. Initially, this seems to contrast with the results of the OHIP-G14; here, the greatest restrictions are indicated after insertion of the aligners. The contradicting results can be explained by the prospect of an esthetically pleasing orofacial appearance. Studies of subjects undergoing orthodontic therapy with fixed multibracket appliances also find an increase in psychosocial well-being based on the PIDAQ [49, 51, 52]. In these studies, however, the subjects experienced a decrease in the subscale “dental self-confidence.” In the case of the present study, the Friedman test yields statistical differences between the survey time points. It can therefore be concluded that therapy with aligners (Invisalign® Teen) in the first year of treatment has a positive effect on the psychosocial well-being of the subjects.

With regard to oral hygiene at home, the test subjects stated that they were more conscientious about it. This is particularly evident in the period from visit 1 to visit 2 and is more noticeable in the male subjects than in the female subjects. The instructions on oral hygiene at home and the professional dental cleanings performed during each visit may have been motivational in this respect. These measures may have contributed to an increased awareness of the importance of home oral hygiene. The Friedman test provides statistical evidence for the reported intensification of oral hygiene at home. Other studies also state improved oral hygiene under orthodontic therapy, both with aligners [53] and with multibracket appliances [54].

The clinical survey on dental plaque accumulation using TMQH provides relatively constant values over the entire survey period. These lie in the lower range of the index—at grade 2 (range 0–5). No significant gender-specific differences are concluded. The Friedman test confirms that there are no statistically verifiable differences between the survey periods. Other studies do conclude increased plaque levels in subjects undergoing therapy with fixed orthodontic multiband appliances [55]. Studies comparing the accumulation of dental plaque during aligner therapy and fixed therapy found lower index values for aligner therapy [24, 56–59].

## Conclusion

Overall, it can be concluded that oral health-related quality of life does not experience statistically provable limitations in adolescents (11 to 17 years) in the first year of therapy with aligners (Invisalign® Teen). Psychosocial well-being increases throughout the course of the treatment. Oral hygiene at home is intensified and there is no clinical evidence of increased dental plaque accumulation. With minor restrictions in the quality of life and increased psychosocial well-being, combined with the prospect of an esthetically pleasing orofacial appearance, sufficient compliance can be assumed. Especially considering the before mentioned aspects and the increased demand of orthodontic treatment in this age range, a therapy with aligners (Invisalign® Teen)—as a less conspicuous orthodontic appliance—should be considered for the central orthodontic patient clientele. This is also supported by the wide range of indications for this treatment method.

## Abbreviations

OHRQoL: Oral Health-Related Quality of Life
DARQoL: Dental Aesthetic Related Quality of Life
OHIP-G14: Oral Health Impact Profile
PIDAQ: Psychosocial Impact of Dental Aesthetic Questionnaire
TMQH: QuigleyHein Plaque Index modified by Turesky et al.
